# Ultrastructural Characterization of the Giant Volcano-like Virus Factory of *Acanthamoeba polyphaga Mimivirus*

**DOI:** 10.1371/journal.pone.0000328

**Published:** 2007-03-28

**Authors:** Marie Suzan-Monti, Bernard La Scola, Lina Barrassi, Leon Espinosa, Didier Raoult

**Affiliations:** Unité des Rickettsies, Centre National de la Recherche Scientifique (CNRS) UMR 6020, IFR 48, Faculté de Médecine, Université de la Méditerranée, Marseille, France; University of Cambridge, United Kingdom

## Abstract

*Acanthamoeba polyphaga Mimivirus* is a giant double-stranded DNA virus defining a new genus, the *Mimiviridae*, among the Nucleo-Cytoplasmic Large DNA Viruses (NCLDV). We used utrastructural studies to shed light on the different steps of the *Mimivirus* replication cycle: entry via phagocytosis, release of viral DNA into the cell cytoplasm through fusion of viral and vacuolar membranes, and finally viral morphogenesis in an extraordinary giant cytoplasmic virus factory (VF). Fluorescent staining of the AT-rich *Mimivirus* DNA showed that it enters the host nucleus prior to the generation of a cytoplasmic independent replication centre that forms the core of the VF. Assembly and filling of viral capsids were observed within the replication centre, before release into the cell cytoplasm where progeny virions accumulated. 3D reconstruction from fluorescent and differential contrast interference images revealed the VF emerging from the cell surface as a volcano-like structure. Its size dramatically grew during the 24 h infectious lytic cycle. Our results showed that *Mimivirus* replication is an extremely efficient process that results from a rapid takeover of cellular machinery, and takes place in a unique and autonomous giant assembly centre, leading to the release of a large number of complex virions through amoebal lysis.

## Introduction

During the environmental study of an outbreak of pneumonia, a giant icosahedral DNA virus was discovered growing in amoebae. This virus was named *Mimivirus* (for *mi*micking *mi*crobe *virus*) [1]. With a diameter of about 650 nm, *Mimivirus* is the largest virus known to date. Morphologically, *Mimivirus* resembles Nucleo-Cytoplasmic Large DNA Viruses (NCLDV), such as the *Iridoviruses, Asfarviruses* and *Phycodnaviruses* [2]. *Mimivirus* comprises a central dense core that is surrounded by two lipid membrane layers inside a capsid protein shell covered by fibrils [3]. The sequence of its 1.2 Mb genome revealed 1262 putative open reading frames (Genbank accession number NC_006450; [4]). A phylogenetic study based on concatenated sequences of the eight class I genes [5] common to *Mimivirus* and to all NCLDVs revealed that *Mimivirus* belonged to this lineage, but stood apart from *Phycodnavirida*e, *Iridoviridae, Asfarviridae* and *Poxviridae* on the phylogenetic tree [4]. In *A. polyphaga, Mimivirus* replicative cycle was described as starting with a 4 h eclipse phase, followed by cytoplasmic accumulation of newly synthesized viruses, and ending with cell lysis and virus release 24 h post-infection (p.i.) [1], [2]. Transmission electron microscopy (TEM) analysis of infected *A. polyphaga* suggested that viral replication, including DNA synthesis and particle assembly, might occur in and near the cell nucleus [1] and the existence of a virus factory was proposed [2]. As already described for a large variety of unrelated viruses, virus factories are perinuclear or cytoplasmic structures where virus replication and assembly take place. Their formation is the result of complex interactions between viral and cellular components and they induce profound alteration of the infected cell structure like recruitment of organelles and organisation of cellular compartments [6]. This paper describes for the first time the morphological characteristics of *Mimivirus* volcano-like giant virus factory, as determined by an extensive ultrastructural study and by tracking fluorescently-labelled viral DNA and viral proteins during the 24 h time course of infection. Our results reinforce the emerging picture of *Mimivirus* as a very complex and unique amoebal pathogen.

## Results

### Ultrastructural aspects of the *Mimivirus* replication cycle

**Figure 1:**
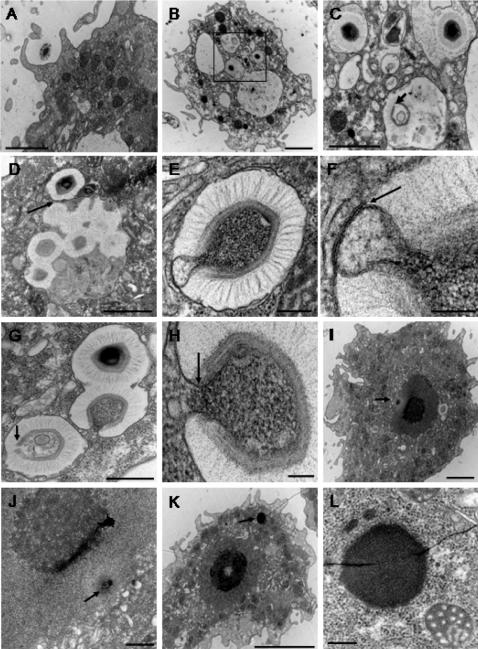
Ultrastructural aspects of the early steps of *Mimivirus* replication cycle. Transmission electron microscopy pictures were taken at 0 h p.i. (A–C) or at 4 h p.i. (D–L). (A) *Mimivirus* particle being phagocytosed by an amoeba; bar = 2 µm. (B) Several single viral particles within intra-cytoplasmic vacuoles; bar = 2 µm. (C) Higher magnification of the boxed area in B showed the open vertex of an empty particle (arrow); bar = 1 µm. (D) Close contact of the membranes of two vacuoles (arrow), one with several *Mimivirus* particles and the other with a single viral particle; bars = 1 µm. (E) Extrusion of the internal *Mimivirus* membrane toward the vacuole membrane; bar = 200 nm. (F) Higher magnification of the contact zone between viral and vacuole membranes (arrow); bar = 100 nm. (G) Full closed, empty with open vertex (arrow) and opening *Mimivirus* particles; bar = 500 nm. (H) Higher magnification of the opening *Mimivirus* particle in G. The fused viral and vacuole membranes were clearly visible (arrow); bar = 100 nm. (I) Condensed electron dense material inside the cell nucleus (arrow); bar = 2 µm. (J) Higher magnification of the condensed electron dense material between the nuclear membrane and the nucleolus (arrow); bar = 500 nm. (K) An electron dense structure (arrow), distinct from the cell nucleus was observed; bar = 5 µm. (L) Higher magnification of this heterogeneous structure, surrounded with mitochondria; bar = 200 nm.

**Figure 2:**
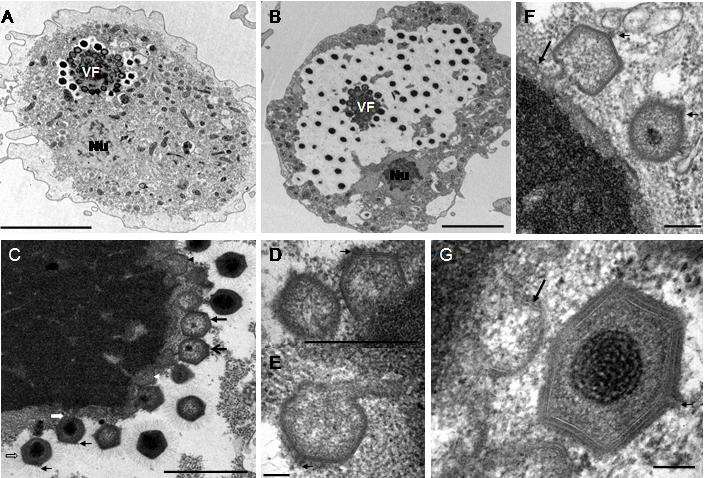
Ultrastructural aspects of the late steps of *Mimivirus* replication cycle - capsid assembly. (A) At 8 h p.i. the virus factory (VF) appeared composed of a dense replication centre surrounded by new viral particles. Nu : cell nucleus; bar = 5 µm. (B) At 12 h p.i. the cell cytoplasm was filled with newly synthesised viruses. The cell nucleus (Nu) was expelled to the periphery; bar = 3 µm. (C–G) Pictures were taken at 8, 12 or 16 h p.i. (C) Different stages of viral particles morphogenesis from the replication centre : beginning of hexagonal capsid assembly (white and black arrowheads); complete empty capsid (thick closed black arrow); filling of empty capsids with condensed electron dense material (thick open black and white arrows); release of full closed viral particles surrounded by fibrils at the periphery of the virus factory; bar = 500 nm. (D–G) Different aspects of viral capsid assembly : beginning of capsid assembly (D bar = 500 nm; G bar = 100 nm); almost complete capsids detaching from the replication center (E bar = 100 nm; F bar = 200 nm); complete capsid being filled with electron dense material (F) or complete viral particle without fibril (G). Membranes were observed beneath the capsid layer (F, G long black arrow). The vertex (small black arrows) was on the external side, opposite to the attachment and filling side (C–G).

*A. polyphaga* were infected with a cell-free *Mimivirus* supernatant at a multiplicity of infection of 10, and processed for TEM at different times p.i.. At 30 min after infection, defined as the 0 h p.i. time point, *Mimivirus* appears to enter the amoebae by phagocytosis (Figure 1A) and was next observed within the phagocytic vacuoles of the amoebae (Figure 1B). Empty particles could be seen with an open vertex (Figure 1C). At 4 h p.i., several viruses could be found within the same vacuole either as fully closed or as empty open particles (Figure 1D and G). The most interesting phenomenon observed was the internal *Mimivirus* membrane extruding from the particle to fuse with the vacuole membrane (Figure 1E and F), and the apparent pouring out of electron dense material, most likely the viral DNA, into the cell cytoplasm (Figure 1G and H). Moreover, these events might occur through the vertex described by Xiao *et al.* [3], since this structure appeared to be open on empty particles (Figure 1C and G). It should be noted that the external structures, the outer layers and fibrils, remained intact on the empty particles at this stage. Next, condensed genetic *Mimivirus* material appears to enter the cell nucleus (Figure 1I and J). These structures were never seen in uninfected amoebae (data not shown). At later p.i. times, we previously described viral particles at the periphery of what we originally thought to be the cell nucleus [1], [2]. Further detailed examination of other series of ultrathin sections of *A. polyphaga* at 4 h p.i. revealed the appearance of an electron-dense structure, clearly distinct from the nucleus that might represent a cytoplasmic viral replication centre surrounded by mitochondria (Figure 1K and L). Contrary to the cell nucleus, this structure did not appear to be surrounded by a membrane. The size of this structure increased rapidly between 5 h and 8 h p.i. At 8 h p.i., newly synthesized viral particles were observed at the periphery of the putative replication centre (Figure 2A), surrounded by an electron-lucent zone, forming a virus factory (VF). At 12 h p.i., almost all the cytoplasmic space was occupied by the VF, and the cell nucleus could still be observed at the periphery (Figure 2B). These observations indicated that *Mimivirus* replication and assembly took place in a very specific cytoplasmic structure composed of a dense central core from which newly formed particles appeared.

The *Mimivirus* factory could be divided into three zones: the inner replication centre, the intermediate assembly zone and the peripheral zone where the newly formed particles acquired their fibrils. This later zone appeared electron-lucent, probably due to exclusion of cellular material and organelles by the expanding VF (Figure 2C, 3A). Closer examination of the VF replication centre suggested a possible sequence of events from assembly of the capsid shell, to the release of complete viral particles with a condensed core surrounded with fibrils. The replication centre of the VF showed a heterogeneous structure with dense inclusions (Figure 2C, 3A). The hexagonal shape of the capsid appeared as assembling progressed (Figure 2C–G), and empty capsids were then filled with electron-dense material before being released (Figure 2C). The vertex was clearly visible on the virus particle, opposite to the side linked to the replication centre (Figure 2C–G). Membranes underlining the capsid layer were observed in growing (Figure 2D–G) and released (Figure 2G) viral particles. These membranes did not encircle the replication centre and were always observed at its periphery. Their origin is still unknown.

**Figure 3:**
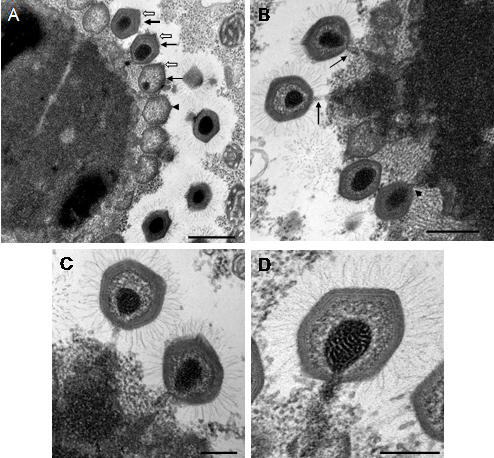
Ultrastructural aspects of the late steps of *Mimivirus* replication cycle – encapsidation of viral DNA. Pictures were taken at 8, 12 or 16 h p.i. (A) Different stages of capsid assembly and DNA encapsidation : complete empty capsid (arrowhead); progressive stages of viral DNA insertion (black arrows) through a portal opposite to the vertex (white arrows); bar = 500 nm. (B) Viral DNA insertion into a capsid (arrowhead) and two different detaching steps of full complete viruses from the replication centre (arrows); bar = 500 nm. (C) Higher magnification of the complete viruses seen in B; bar = 200 nm; (D) Insertion of condensed viral DNA into a viral capsid; bar = 200 nm.

Figure 3 illustrated how viral DNA might be encapsidated into nascent *Mimivirus* particles. The encapsidation process occurred at the replication centre periphery once capsid assembly is almost complete (Figure 3A). Viral DNA condensation seemed to begin within the replication centre (Figure 3D) before being inserted into viral capsids through an open portal located on the opposite side to the vertex (Figure 3B–D). Figure 3 C and D showed how viral DNA condensation progressed within the viral capsid to form the core centre of the viral particle.

Altogether these observations compelled us to modify our original interpretation and to further characterize the different stages of the *Mimivirus* assembly pathway.

### Morphological description of the *Mimivirus* factory

**Figure 4:**
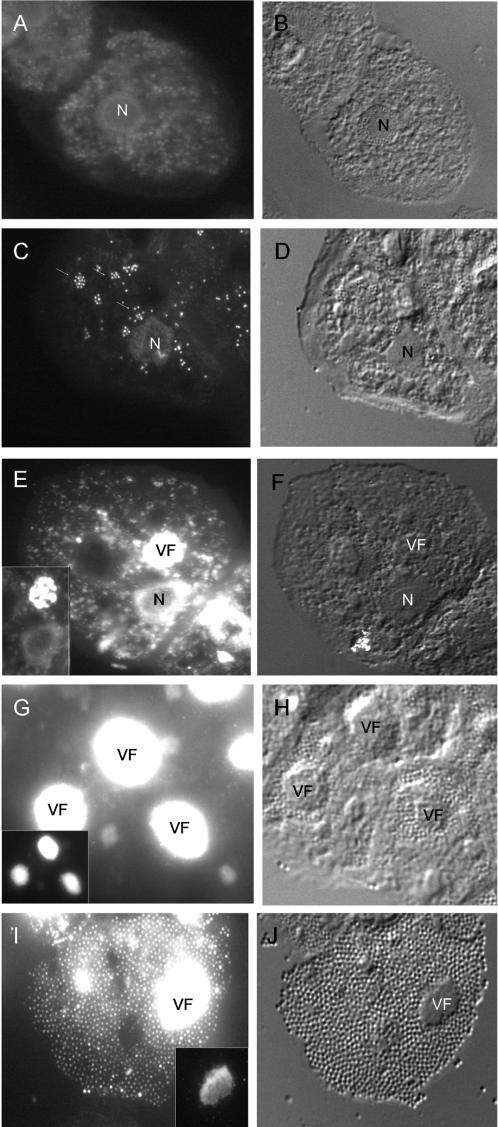
Mimivirus infectious cycle. *Mimivirus* infected *A. polyphaga* were stained with DAPI at different times points p.i. and representative pictures are shown. A, B : non infected amoebae; C, D: 0 h p.i. *Mimivirus* particles inside the cytoplasm and near the cell nucleus could be seen; E, F: 4 h p.i. The heterogeneous structure of the VF appeared near the cell nucleus. No viral particles were detectable in the cytoplasm; G, H : 8 h p.i. The intensively stained VF appeared as an homogeneous structure and neosynthesized viral particles accumulated around the VF; I, J : 18 h p.i. The VF was still intensely stained with quite a different structure, whereas the cell cytoplasm was completely filled with new viral particles. Fluorescence (left column) and DIC (right column) images of the same slide field were taken with a 63×/1.4 oil lens. Fluorescence pictures were taken with an exposure time of 1 sec (A) and 64 msec with gain 2 (C, E, G, I). Inset pictures corresponded to the same as E, G and I taken at a different exposure time 64 msec (E, G) and 16 msec respectively (I).

The formation kinetics of the VF, and its viral DNA content in particular, were studied by direct fluorescent staining with the blue fluorescent stain DAPI. The choice of this molecule was based on the fact that DAPI preferentially stains dsDNA by association with AT clusters in the minor groove [7]. The *Mimivirus* genome has a high AT proportion (72%, [4]) compared to *A. polyphaga* (genomic AT content estimated to be 49%, determined using 96 shotgun sequences of amoebae genomic DNA; data not shown). *Mimivirus*-infected *A. polyphaga* were consequently stained at different time points p.i. Fluorescence and differential interference contrast (DIC) images of the same field are presented in Figure 4. Representative images are also shown in the Supporting Information (Text S1, Figure S1). In uninfected amoebae, cell nuclei showed a characteristic ring-like staining pattern with unlabeled nucleoli surrounded with labelled chromatin, similar to the nucleus morphology observed with TEM (Figure 4A and B). At 0 h p.i. *Mimivirus* nucleic acid staining appeared as bright single or clustered dots within the cell cytoplasm, contrasting with the cytoplasmic background DAPI labelling (Figure 4C and D). At 1 h p.i., the *Mimivirus* DAPI-stained DNA dots reached the cell nuclei, where size and staining intensity increased until 3 h p.i. At 4 h p.i. strongly stained clusters showing a heterogeneous structure appeared outside of the cell nuclei (Figure 4E and F). In most of the cells showing these structures, only one cluster could be seen per infected amoeba. Similar observations were made using standard DNA-staining histological dyes such as carbolic toluidine blue (data not shown). Such structures were not observed in uninfected amoebae. The size of these clusters peaked between 8–12 h p.i. and sustained their maximal size and staining intensity until the end of the replication cycle. However at 8 h p.i. the clusters exhibited a homogeneous structure (Figure 4G and H), whereas at 18 h p.i., they showed a heterogeneous less organised morphology. The time course of the development of these structures and their morphology clearly showed that they corresponded to the replication centre of the VFs observed by electron microscopy.

**Figure 5:**
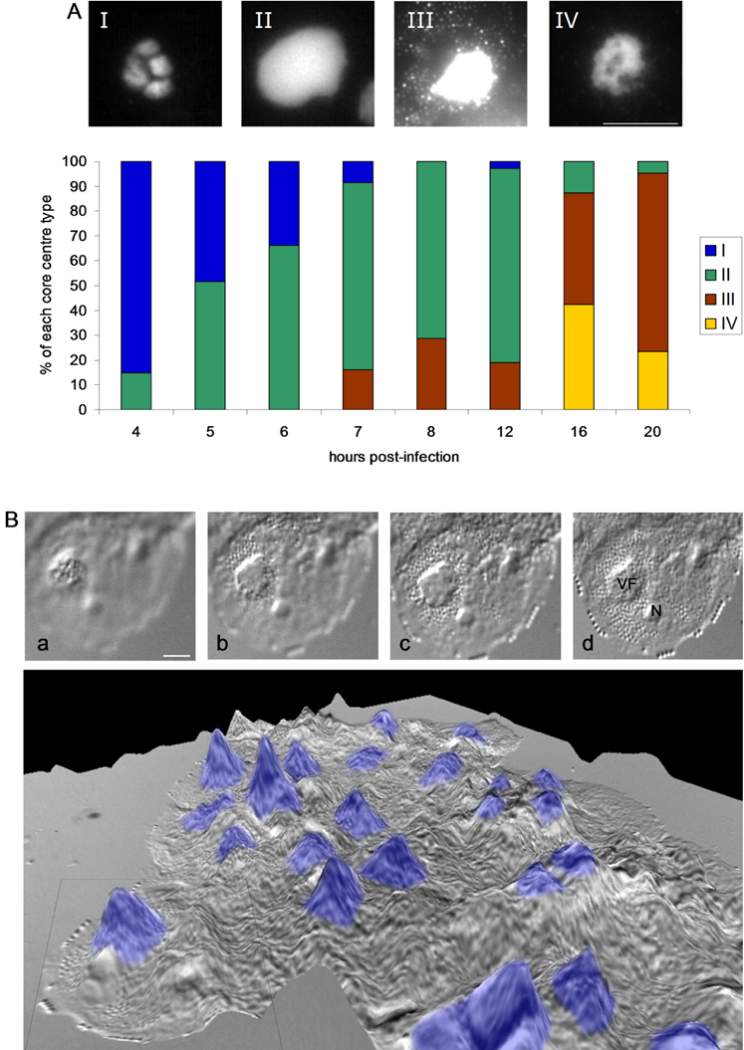
Evolution and 3D reconstruction of *Mimivirus* factory. A: Characterisation and quantification of the different types of replication centre. Fluorescence pictures were taken at 4 h (I, exposure time 32 msec), 8 h (II and III, exposure time 64 and 128 msec respectively) and 16 h (IV, exposure time 32 msec) p.i. Bar = 10 µm. Histogram : a total of 717 DAPI-stained cells were analysed to quantify the proportion of each replication centre time at the indicated time points. B: DIC pictures of an *APM* infected *A. polyphaga* at 8 h p.i. taken with a 63×/1.4 oil lens. Different sections according to the depth of focus are shown downward (upper part, from a to d). Bar = 10 µm. The lower part represented a 3D reconstruction combining DIC and DAPI staining of the VF present in different infected cells of a microscope field. The dotted line box framed the cell analysed in the upper part. Blue : DAPI-staining.

Because our experimental conditions used non-synchronized infected cells, we quantified the proportion of each of these morphologically distinct types of replication centre at different time points p.i. in order to determine whether there might be a progression from one type to the other across the time course of infection. Results are shown in Figure 5. Four different core centre morphologies were characterised and quantified (Figure 5A) : i) type I with a clustered morphology (see also Figure 4E), predominant in the first 4 hours of infection; ii) type II in which the core centre appeared as a completely homogeneous structure with a blurry aspect in microscopy images (see Figure 4G), most likely resulting from the fusion of the clusters seen earlier. This form was predominant from 6 to 12 h p.i.; iii) type III in which the whole *Mimivirus* DNA cluster was surrounded by more and more small bright dots, similar to virus particles, quickly filling the cytoplasmic volume. This form was detected from 7 h p.i. to the end of the infection; and iv) type IV in which the clusters had a heterogeneous morphology with a disorganized appearance with holes and fiber-like patterns (see also Figure 4I). This form was detected in the latest times of infection. These results allowed us to propose a progression of the different characteristic *Mimivirus* production stages from an early heterogeneous stage (I) corresponding to the appearance and formation of the VF core centre, followed by a homogeneous “mature” stage (II) corresponding to the growing core centre, then by a “productive” stage of the VF (III), and finally by a heterogeneous “degenerative” stage (IV) which most probably signed the exhaustion of the *Mimivirus* factory. Confocal data were used to build 3D volumic reconstruction of the three main types of *Mimivirus* factory replication centre during the time course of infection (see also Supporting Information Text S1, Figure S2A). Variation of the DAPI intensity staining is indicative of the variation of the DNA content in the core centre. A quantitative analysis is presented below.

### Specific aspects of the volcano-like giant *Mimivirus* factory

The height of the cells during the late phase of infection, and the limited depth of focus of the 63× objective, allowed us to explore their 3D organisation based on DIC images. Details from one cell are shown in the upper part of Figure 5B. The maximum height of the cell was estimated to be about 10 µm using the difference between the uppermost and the lowest focused images that could be obtained (Figure 5B, sections a and d, respectively); this measurement was confirmed using confocal optical slides. The uppermost focused image always corresponded to the top of the VF as identified by DAPI staining (section a). Intermediate images (sections b and c) showed the newly synthesized virus particles spreading all around the VF and finally organizing into a single-layer crystal-like structure at the bottom of the cell (section d, see also Figure 4J). The Extended Depth of Focus (EDF) technique [8] allowed us to generate a topological view of all regions on the same focal plane. EDF allowed us to build a 3D image of the volcano-like structure found in *Mimivirus*-infected cells as shown in Figure 5B, lower part. The position of the VF replication centre in the EDF image was obtained by the overlay of the DAPI fluorescence image. The area (µm^2^) of the VF increased from 110 µm^2^ at 4 h p.i. to 250 µm^2^ at 12 h p.i. which represented about 42% of the cell surface (data not shown). These characteristics classified the *Mimivirus* factory among the largest described until now.

All the results obtained by transmission electron microscopy, widefield fluorescence, confocal and volume reconstruction analyses allowed us to propose a 3D model of the morphology of *Mimivirus* factory (Supporting Information, Figure S2 B).

### Fluorescence intensity quantification

**Figure 6:**
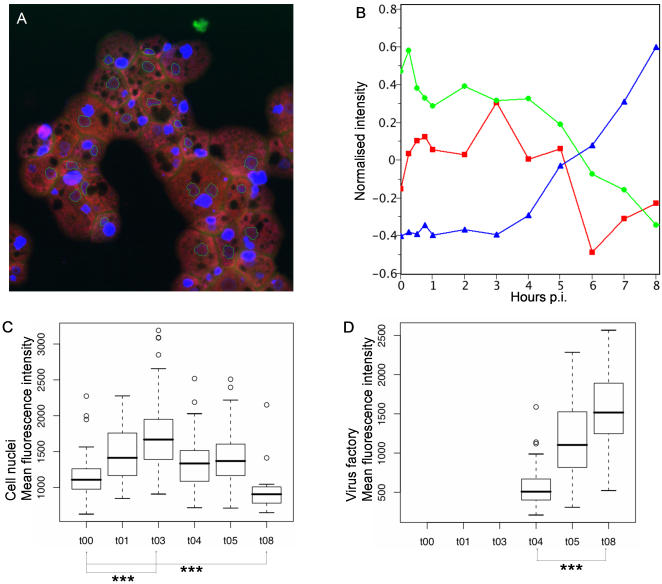
Quantification of the kinetics of *Mimivirus* factory formation. (A) Fluorescence picture of a representative field from *A. polyphaga* at 5 h p.i., stained with DAPI (40× magnification/0.7 lens). The nuclei are marked around their edge with a blue line to allow quantification of their areas compared to those of the *Mimivirus* factories. (B) Quantification curve showing the normalized parameter (y-axis) as a function of time p.i. (x-axis). Red line: nuclei intensity; green line: nuclei surface fraction; blue line: *Mimivirus* factory surface fraction. Evolution of the mean fluorescence intensity over time p.i. in cell nuclei (C) or in virus factories (D), at 100 and 4 msec exposure time respectively, using R software; *** = p<0.01 (Wilcoxon test).

**Figure 7:**
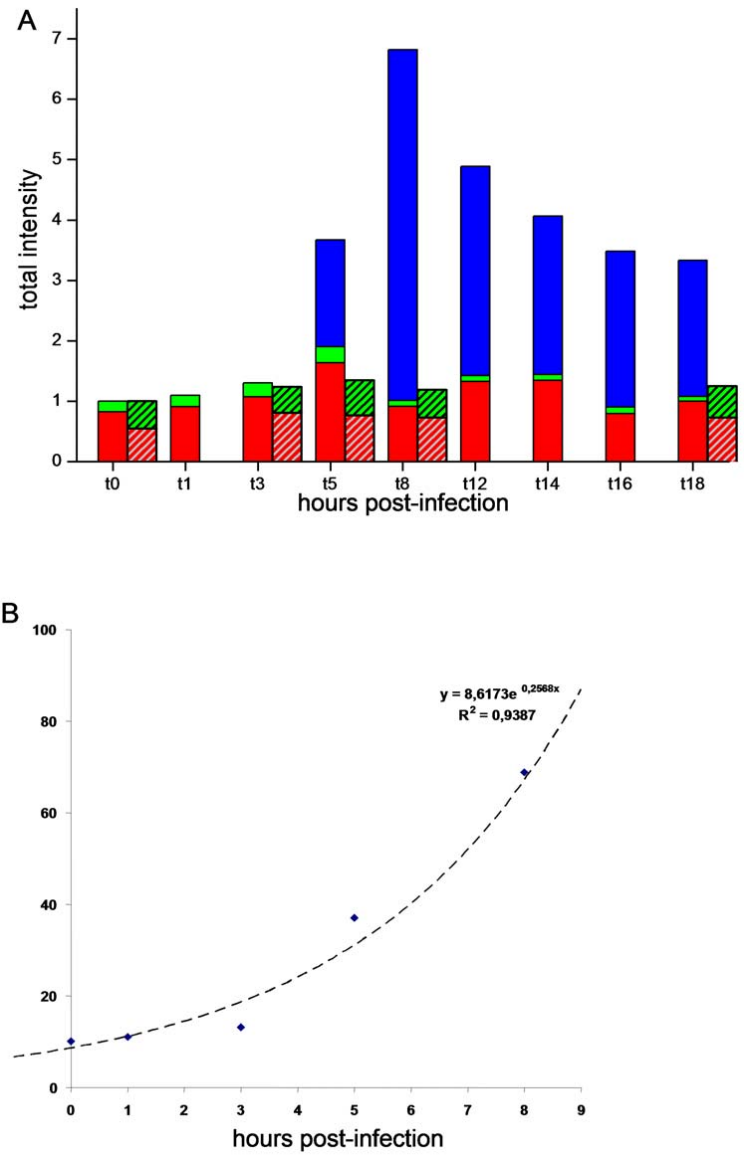
Increase in total cellular DNA content in *A. polyphaga* during the *Mimivirus* infection cycle. A. Estimation of the total amount of cellular DNA by fluorescence intensity quantification of DAPI staining in *Mimivirus* infected (bars) or uninfected amoebae (hatched bars). Total intensity = staining area (pixels) × mean intensity (intensity/pixel). The total intensity at different time points was divided by the intensity at time = 0 h p.i. for normalization. The bar height represents the variation of DNA content compared to the t0 timepoint for different cellular compartments. Red bar: nucleus; green bar: cytoplasm; blue bar: virus factory. B. The increase of total cellular DNA was extrapolated from 0 to 8 h p.i. and fitted with the exponential equation: y = A0.e^(Rx)^. Data showed a high predictability with the exponential growth model (R2 = 0.94).

During the infection cycle, nuclear DAPI staining peaked from 0 to 3 h p.i. and then decreased. After 8 h p.i., nuclei showed weaker labelling and a modified appearance: newly synthesized viral particles could be observed as single dots in the cytoplasm. The number of dots increased dramatically by the end of the replication cycle, at which time they completely filled the intracellular space. One interpretation of these results is that during the 0–3 h p.i. period, the increased nuclear fluorescence intensity was the consequence of the transient nuclear localization of *Mimivirus* DNA, which then moved into the cytoplasm to form the highly fluorescent VF. The brightness of the VF fluorescence indicated the accumulation of AT-rich DNA (Figures 4 and S1). Fluorescence intensity was analyzed and quantified as described in the Materials and Methods section, and results are shown in Figure 6. The fluorescence attributes (mean intensity and area) of nuclei and *Mimivirus* factory showed a concomitant and inverse evolution compared to each other, with the most remarkable point around 5 h p.i.: at this time, the cell area occupied by nuclei showed a 50% drop, whereas the cell area occupied by the *Mimivirus* factory showed a 50% increase (Figure 6 A and B). Statistical analysis revealed a significant increase of the mean nuclear fluorescence intensity between 0 h and 3 h p.i. (p<0.01) and a significant decrease between 0 h and 8 h p.i. (p<0.01; Figure 6 C). Conversely, there was a significant increase in the mean VF fluorescence intensity between 4 h and 8 h p.i. (p<0.01; Figure 6 D). Taken together, these observations favour a model in which the major site of *Mimivirus* DNA replication is the cytoplasmic VF, and further suggest that there is a relationship between the two different structures during the replication cycle. Quantification of total fluorescence intensity at different time points p.i. showed an 7-fold increase in total DNA in the cell between 0 h and 8 h p.i., which is exponential growth (e^0.2568x^, Figure 7), equivalent to a doubling time of 2.7 h. In comparison, the total fluorescence intensity in uninfected amoebae varied from 1 at 0 h to 1.2 at 8 h (Figure 7, hatched bars). These results complemented the microscopy results, and allowed the first insights into *Mimivirus* replication cycle.

## Discussion

**Figure 8:**
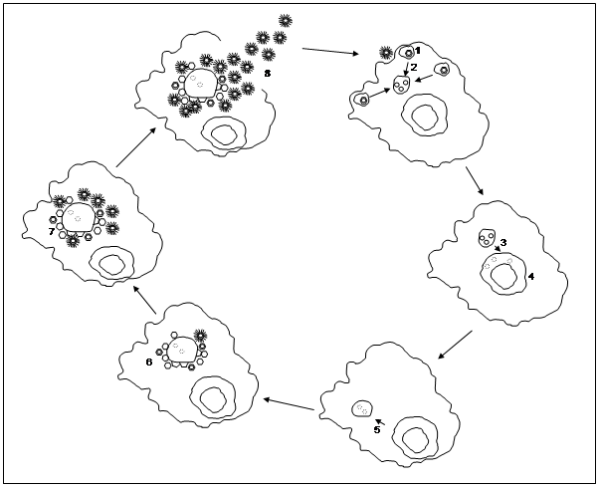
Schematic representation of *APM* replication cycle. *Mimivirus* entry through a phagocytic vacuole (1). Fusion of phagocytic vacuoles (2) and delivery of *Mimivirus* genetic material into the cell cytoplasm (3). *Mimivirus* DNA entry into the host nucleus (3), where the first round of DNA replication might begin (4). At 3 h p.i. *Mimivirus* DNA came out the host nucleus to form the VF replication centre (5). At 5 h p.i. the VF size showed a 50% increase and viral proteins began to be detected. Proviral capsid assembly and viral capsids budding from the VF central core could be observed (6). Empty or DNA filled capsids accumulated nearby the central core, resulting in a growing VF with viral particles free in the cytoplasm (7). Complete viral capsids surrounded by fibrils might be released through cell lysis (8).

*Mimivirus* is a pathogen resistant to phagocytic destruction in amoebae, and as such should be considered as a possible new causative agent of human pneumonia. Indeed, links with human pneumonia were recently reported [9]–[11]. Among the different amoebae-resistant pathogens studied to date, *Mimivirus* appears to be the only one with such a rapid lytic effect on amoebae [12]. Until now, little was known about the different steps of the *Mimivirus* replication cycle. Our initial electron microscopy observations of *in vitro* *Mimivirus*-infected *A. polyphaga* showed the intra-cytoplasmic production and accumulation of newly synthesised viruses within a 24 h lytic cycle [1], [2]. In these papers, we initially speculated that *Mimivirus* multiplied in the nuclei of infected cells. Indeed, we mistakenly identified the host nucleus as the VF because of its size and aspect. In the present study, in addition to ultrastructural characterization, the unusual size of *Mimivirus* allowed us to follow the different stages of its replication cycle using fluorescence and DIC microscopy. This enabled us to characterize the formation and growth of the giant *Mimivirus* VF, and to describe how progeny virions are synthesised, assembled and released from the replication centre to invade the cytoplasmic space. We propose the following replication cycle (Figure 8), composed of an early phase between 0–3 h p.i. (steps 1–4) and of a late phase thereafter (steps 5–8).

Electron microscopy images indicated that *Mimivirus* entry into the amoebae was most likely due to a phagocytic process, followed by fusion of phagocytic vacuoles. *Mimivirus* genetic material was delivered into the cytoplasm at this stage after fusion of the viral and vacuole membranes, most probably through the virus vertex. Whether the *Mimivirus* vertex plays also a role in the attachment to the cell surface, as described for Phycodnaviruses [13], [14], remains to be established. Quantification from fluorescent nucleic acid labelling studies between 0 h and 3 h p.i. showed an increase in intensity in the amoebae nucleus, reflecting an increase in AT-content that might be the result of *Mimivirus* DNA acquisition. We hypothesise that *Mimivirus* DNA first enters the amoebae nucleus, shortly after infection, probably for a first round of replication. At 3 h p.i., AT-rich *Mimivirus* DNA becomes localised to the cell cytoplasm in a structure distinct from the cell nucleus. This might be interpreted as the exit of the *Mimivirus* genetic material from the nucleus to form the replication centre of the VF in the cell cytoplasm. This structure has also been observed in ultrastructural studies, and it may be the major site of *Mimivirus* DNA production, independent of the cell nucleus machinery. At 8 h p.i., transmission electron microscopy, direct and indirect fluorescence labelling and quantification of *Mimivirus* DNA or protein allowed us to clearly distinguish the *Mimivirus* factory from the cell nucleus. This stage was characterized by an increase in viral DNA production within the cytoplasmic VF, while DNA staining in the host nucleus decreased. The *Mimivirus* factory size increased with time and with the production of progeny virions. This datasuit may be illustrated by a 3D model of the *Mimivirus* factory, composed of a replication centre made of unpackaged DNA, all around which viruses are formed in an assembly zone before being released in the cell cytoplasm (Figure S2B).

The present results indicate that pre-formed capsids are filled with viral DNA, since all of the successive steps of capsid formation could be observed. As viral capsids were shown to assemble at the periphery of the replication centre, it might be envisaged that viral proteins are partially or fully concentrated or synthesised in the replications centre; this idea is supported by results obtained with a *Mimivirus*-specific mAb recognizing the late virion-associated R710 protein (Supporting Information Text S1, Figure S3). Furthermore, proteomic data analysis showed that no cellular host proteins seem to be incorporated within the virus particles [15] which might be indicative of an active mechanism of cell protein exclusion. It is not known whether gene extinction and cell machinery hijacking occurs in the *Mimivirus* factory to allow its replication and production, as has been described for other VFs [6].

A large variety of virus factories have been described for unrelated viruses [6]. It has previously been demonstrated that the replication site is predominantly cytoplasmic for *Poxviridae* [16], nuclear and cytoplasmic for *Asfarviridae* [17], [18] and nuclear for *Iridoviridae* and *Phycodnaviridae* [13], [14], [19], [20], whereas the assembly sites are all cytoplasmic. The main characteristics of these viruses, as well as their replication and assembly sites, are summarized in Supporting Information, Table S1. Here, we described a new VF, which might be specific to the *Mimiviridae*, with a still undescribed replication centre which may insure a high degree of replication autonomy for this virus family regarding the host cell machinery. Similarities to the *Asfarviridae* could be observed, particularly in terms of early nuclear viral DNA replication, DNA insertion/encapsidation into pre-formed capsids, and number of VF per infected cell [17], [21]. However major differences are noticeable such as the weak detection of membranes within the VF [22], or the larger *Mimivirus* factory area compared to ASFV [17]. This is also true when comparison is made with other large DNA viruses factories which may occupy a large region of the infected cell [6]. Another difference is the absence of membrane surrounding the Mimivirus factory. Several questions are raised by our results. First, what is the source of nucleotides for building such a large DNA structure? Second, is there an exploitation of the aggresome pathway by cytoplasmic *Mimivirus* DNA to concentrate viral proteins at the assembly site, as previously reported for African swine fever virus [23]? Third, how are host proteins and organelles excluded from the VF region, and how is the cellular cytoskeleton reorganized?

In conclusion, the *Mimivirus* particle, composed of RNA transcripts combined with more than 100 viral proteins, appears to be particularly complex. Very specific mechanisms and complex interactions between viral and cellular factors must be involved to build this remarkably large and efficient VF, which can rapidly generate such a sophisticated microorganism.

## Materials and Methods

### Viral infection

*A. polyphaga* were seeded at 4×10^5^ cells/ml in Page's amoebal saline (PAS) [24], infected with titrated *Mimivirus* at an amoeba cell:virus ratio of 1∶10 and centrifuged at 1,000× g for 30 min. Amoebae viability was estimated by counting the cells immediately after centrifugation and every two hours after that for the next 32 h.

### Electron microscopy and immunofluorescence

*Mimivirus*-infected *A. polyphaga* were prepared for TEM as follows. Cells were washed three times in PBS, resuspended in 5% glutaraldehyde (Sigma) in PBS for 1 h at 4°C and then washed again three times in PBS. The cell pellet was fixed in 1% osmic acid, washed twice in PBS, dehydrated in 50, 70, 95 and 100% alcohol and embedded in Epon.

For fluorescence labelling, 100 µl of cell suspension at 4×10^5 ^cells/ml were put into a Cytospin chamber, centrifuged for 10 min at 800 rpm in a Shandon Cytospin 4 (Thermo Electron Corporation) and then fixed for 10 min in methanol. For direct fluorescence with DAPI (4′,6′-diamidino-2-phenylindole) staining, cells were covered with 5 µM DAPI from a ready-to-use solution, “ProLong Gold Antifade Reagent” (Molecular Probes) and stained for 10 min in the dark prior to observation. For indirect immunofluorescence, 100 µl of mAb P4C8G2, raised against purified *Mimivirus* (data not shown), was diluted 1∶100 in PBS with 3% (w/v) non-fat dry milk and added to the slides. Slides were incubated in a moist chamber at 37°C for 30 min. After three washes in PBS, the slides were incubated for 30 min at 37°C with 100 µl of a FITC-conjugated goat anti-mouse Ig (Jackson ImmunoResearch) diluted 1∶100 in PBS containing 0.2% Evans blue. After three washes with PBS, the slides were mounted using a phosphate-buffered glycerol medium, pH 8, prior to observation.

Cells were observed using upright microscopes (Olympus BX 51and Zeiss Axio Imager) equipped with 40×, 63× or 100× lenses. DIC images were acquired using the Axio Imager microscope. All images were acquired with a cooled (−30°C) DS1-QM (Nikon) black and white camera driven by “Lucia G” software (Nikon & LIM Ltd. Prague, Czech Republic). DAPI-fluorescence images were taken using a DAPI filter (360/55 nm; 460/50 nm). FITC-mAb images were taken using an FITC filter (480/20; 535/40). Confocal images were acquired with an LSM 510 Zeiss microscope, with DAPI staining observed using a UV diode (405 nm), z step = 0.3 µm. 3D volumic reconstruction was achieved using OsiriX Medical Imaging Software [25]. The topology of infected cells was obtained using limited depth focus DIC images. The 3D reconstruction was obtained with Lucia software's EDF algorithm. The position of the VF replication centre in the EDF image was obtained by overlay of the DAPI fluorescence image.

Image analysis was performed using “Lucia G” and ImageJ software (Rasband, W.S., ImageJ, National Institutes of Health, Bethesda, Maryland, USA, 1997–2006, http://rsb.info.nih.gov/ij/). Images were acquired in 12 bit depth with the same exposure parameters. Three images per field were recorded, and a total of 1030 cells were analyzed using the following protocol. The absolute value of intensity was measured in regions of interest (ROIs): nucleus, cytoplasm, DNA clusters, monoclonal Ab staining, background. The following parameters were measured: area = sum of ROIs area; area fraction = area/area of cells in the field; intensity = [mean of (ROIs intensity/ROI area)]–background. In order to compare in the same graph the variations of the parameters having different units (Intensity, Area Fraction) we calculated for each parameter the “centered and normalized” value using the formula: X_t_ = (X_t_−mean_X_)/(X_max_−X_min_).

To quantify the evolution of the mean fluorescence intensity, measurements were compared: 0 h p.i. versus 3 h p.i. and 0 h p.i. versus 8 h p.i. For this purpose, four parameters were measured in DAPI-stained *Mimivirus* infected cells: nuclear area, mean nuclear fluorescence intensity, VF area and mean VF fluorescence intensity. For fluorescence quantification, acquisition time was 4 msec for the VFs and 100 msec for the nuclei. Statistical analyses were performed with the Wilcoxon test using R software (R Development Core Team (2006). R: A language and environment for statistical computing. R Foundation for Statistical Computing, Vienna, Austria. ISBN 3-900051-07-0, URL http://www.R-project.org.)

## Supporting Information

Text S1 (0.04 MB DOC)

Table S1 General characteristics of virus factories of large dsDNA viruses
(0.04 MB DOC)

Figure S1 Kinetics of Mimivirus factory formation. The cellular location of Mimivirus AT-rich DNA was monitored by DAPI staining during the time course of A. polyphaga infection. Fluorescence images were taken with a 40× lens with an exposure time of 64 msec (main images) or with a 63×/1.4 oil lens with an exposure time of 128 msec (inset images).
(0.35 MB TIF)

Figure S2 3D reconstruction and model of Mimivirus factory. (A) Volumic reconstruction of Mimivirus factory. DAPI stained Mimivirus infected A. polyphaga were observed with a confocal microscope at 16 h p.i. Fluorescence intensity was represented by a rainbow logarithmic look up table. The respective 2D maximum intensity projections of the II, III and IV regions are shown in the lower part : II, mature stage of the growing VF; III, productive stage; IV, degenerative stage. Bar = 10 µm. (B) 3D model of Mimivirus factory.
(0.31 MB TIF)

Figure S3 Molecular characterization of the Mimivirus factory. Combined labelling of Mimivirus AT-rich DNA with DAPI staining (direct fluorescence; blue) and Mimivirus R710 protein with a specific mAb by indirect immunofluorescence (green) was performed during the time course of A. polyphaga infection. The protein showed a punctuated staining pattern starting at 6 h post-infection around the DAPI-stained Mimivirus factory. Thereafter, the number and intensity of anti-R710 mAb-stained Mimivirus factory increased until the end of the viral cycle.
(0.76 MB TIF)
