# Supplementary material for: Ultrastructural Characterization of the Giant Volcano-like Virus Factory of Acanthamoeba polyphaga Mimivirus
Source: PLoS One. 2007 Mar 28;2(3):e328. doi: 10.1371/journal.pone.0000328 (PMC1828621; doi:10.1371/journal.pone.0000328)
Supplement: Text S1 — (0.04 MB DOC) [file pone.0000328.s001.doc]

**Supporting informations**

**Kinetics of *Mimivirus* factory formation.** The cellular location of *Mimivirus* AT-rich DNA was monitored by DAPI staining during the time course of *A. polyphaga* infection (Figure S1). At early time points, 0 and 1 h p.i., *Mimivirus* DNA appeared as single or clustered dots within the cytoplasm; concurrently, the intensity of nuclear fluorescence increased until 3 h p.i. By this time, brightly stained clusters distinct from the nuclei could be observed. From 5 h p.i., the number, surface area and fluorescence intensity of these clusters increased until the end of the viral cycle. At 8 h p.i., newly formed viral particles could be seen in the cytoplasm, to finally occupy the majority of the cell surface at 17h p.i.

**Morphological description of the *Mimivirus* factory**

Since we used non-synchronised infected cells, confocal analysis was performed on DAPI-stained cells at 16 h p.i. to reveal the internal structure of *Mimivirus* VF followed by 3D volumic reconstruction (Figure S2 A). In order to show the different regions inside the VF, the fluorescence intensity was represented by logarithmic look up table. The three main stages of *Mimivirus* VF evolution during the time course of infection were observed. They were named according to their description by fluorescence and DIC analysis (Figures 4 and 5A) : type II, a big and spheric VF defined as a "mature state" that shows a very intense and homogeneous DNA core surrounded by a thiny less bright "cortex"; type III, a VF in a "productive state" that has irregular shape,  a thicker "cortex" and an even less bright peripheral region composed by small particles; type IV, a VF in a degenerative state which lost the homogeneous and intense DNA core centre.

All the results obtained by transmission electron microscopy, widefield fluorescence, confocal and volume reconstruction analyses allowed us to propose a 3D model of the morphology of *Mimivirus* factory (Figure S2 B).

**Immunofluorescence labelling of the *Mimivirus* factory**

Since DAPI staining is not strictly specific for *Mimivirus* DNA, we wished to further characterize the *Mimivirus* factory. We thus combined DAPI-staining and indirect immunofluorescence labelling, using a specific monoclonal antibody (mAb) P4C8G2 raised against purified *Mimivirus* (unpublished data). The protein recognized by this mAb is coded for by ORF R710. It is a 194-amino acid protein of unknown function with an apparent 21.9 kDa molecular weight (unpublished data). Results are shown in Figure S3. Little or no labelled R710 protein was seen at the early time points (0 h and 1 h p.i.). At 6 h p.i., a punctuated labelling pattern was seen around the DAPI-stained *Mimivirus* factory, suggesting that R710 is a late expressed protein. After 12 h p.i., the intensity of R710 labelling as well as the number of labelled and stained *Mimivirus* factories increased, and by the end of the replication cycle (16 h p.i.) all the *Mimivirus* factories showed double staining. These results demonstrated that these structures contain viral protein as well as DNA, and suggest that within the giant VF, both *Mimivirus* nucleic acids and proteins might be produced or concentrated to allow the assembly and morphogenesis of virion progeny.

**Legends to figures**

**Figure S1. Kinetics of *Mimivirus* factory formation.** The cellular location of *Mimivirus* AT-rich DNA was monitored by DAPI staining during the time course of *A. polyphaga* infection. Fluorescence images were taken with a 40 x lens with an exposure time of 64 msec (main images) or with a 63x/ 1.4 oil lens with an exposure time of 128 msec (inset images).

**Figure S2. 3D reconstruction and model of *Mimivirus* factory.** (A) Volumic reconstruction of *Mimivirus* factory. DAPI stained *Mimivirus* infected *A. polyphaga* were observed with a confocal microscope at 16 h p.i. Fluorescence intensity was represented by a rainbow logarithmic look up table. The respective 2D maximum intensity projections of the II, III and IV regions are shown in the lower part : II, mature stage of the growing VF; III, productive stage; IV, degenerative stage. Bar = 10 µm. (B) 3D model of *Mimivirus* factory.

**Figure S3. Molecular characterization of the *Mimivirus* factory.** Combined labelling of *Mimivirus* AT-rich DNA with DAPI staining (direct fluorescence; blue) and *Mimivirus* R710 protein with a specific mAb by indirect immunofluorescence (green) was performed during the time course of *A. polyphaga* infection. The protein showed a punctuated staining pattern starting at 6 h post-infection around the DAPI-stained *Mimivirus* factory. Thereafter, the number and intensity of anti-R710 mAb-stained *Mimivirus* factory increased until the end of the viral cycle.
