# Supplementary material for: Ultrastructural Characterization of the Giant Volcano-like Virus Factory of Acanthamoeba polyphaga Mimivirus
Source: PLoS One. 2007 Mar 28;2(3):e328. doi: 10.1371/journal.pone.0000328 (PMC1828621; doi:10.1371/journal.pone.0000328)
Supplement: Table S1 — General characteristics of virus factories of large dsDNA viruses (0.04 MB DOC) [file pone.0000328.s002.doc]

**Table S1. General characteristics of virus factories of large dsDNA viruses**

| Virus family  (number of members) | Representative virus | Genome structure  (size) | Number of proteins  (number in virions) | Virion structure | Replication site | Assembly site |
| --- | --- | --- | --- | --- | --- | --- |
| *Poxviridae*^a^  (>100) | Vaccinia | dsDNA  (130-375 kbp) | 150-300  (~100) | enveloped, brick-shaped  >300 nm | cytoplasmic, RER-associated | cytoplasmic membranes |
| *Asfarviridae*^a^  (1) | ASFV* | dsDNA  (170-190 kbp) | ~150  (~50) | enveloped, icosahedral  175-215 nm | nuclear and cytoplasmic | cytoplasmic membranes |
| *Iridoviridae*^a^  (>20) | IIV-6* | dsDNA  (140-303 kpb) | >100  (>36) | enveloped, icosahedral  120-350 nm | nuclear | cytoplasmic |
| *Herpesviridae*^a^  (>130) | HHV-1* | dsDNA  (125->240 kbp) | 70->200  (>30) | enveloped, icosahedral capsid  125 nm | nuclear | nuclear and cytoplasmic |
| *Phycodnaviridae*^b^  (>25) | PBCV-1* | dsDNA  (300-380 kbp) | ~376  (~50) | enveloped, icosahedral capsid  (140-190 nm | nuclear | cytoplasmic |
| *Mimiviridae*  (1) | APM* | dsDNA  (1181 kbp) | 911  (~100) | icosahedral capsid  ~650 nm | transiently nuclear then cytoplasmic | cytoplasmic |

*ASFV : african swine fever virus; IIV-6 : invertebrate iridescent virus 6; HHV-1 : human herpes virus 1; PBCV-1 : *Paramecium bursaria* chlorella virus 1; APM : *Acanthamoeba polyphaga* Mimivirus.

a : data from ref. [6] ); b : data from ref. [26].
