# Supplementary figures and images for: Ultrastructural Characterization of the Giant Volcano-like Virus Factory of Acanthamoeba polyphaga Mimivirus
Source: PLoS One. 2007 Mar 28;2(3):e328. doi: 10.1371/journal.pone.0000328 (PMC1828621; doi:10.1371/journal.pone.0000328)

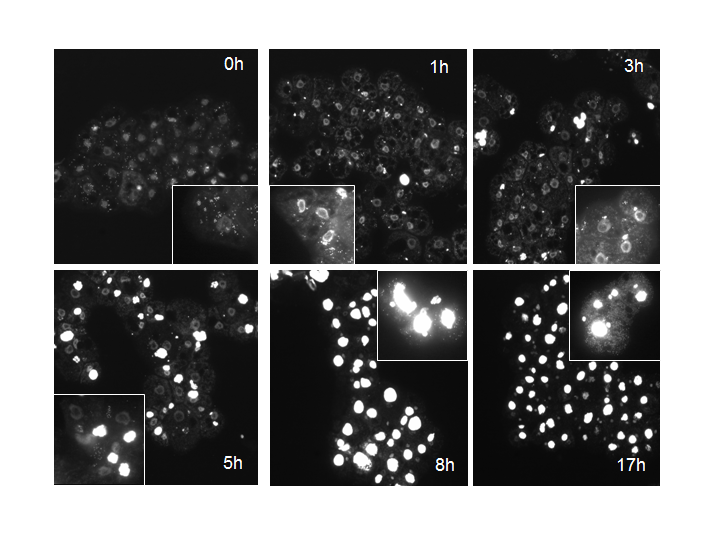

Supplement: Figure S1 — Kinetics of Mimivirus factory formation. The cellular location of Mimivirus AT-rich DNA was monitored by DAPI staining during the time course of A. polyphaga infection. Fluorescence images were taken with a 40× lens with an exposure time of 64 msec (main images) or with a 63×/1.4 oil lens with an exposure time of 128 msec (inset images). (0.35 MB TIF) [file pone.0000328.s003.tif]

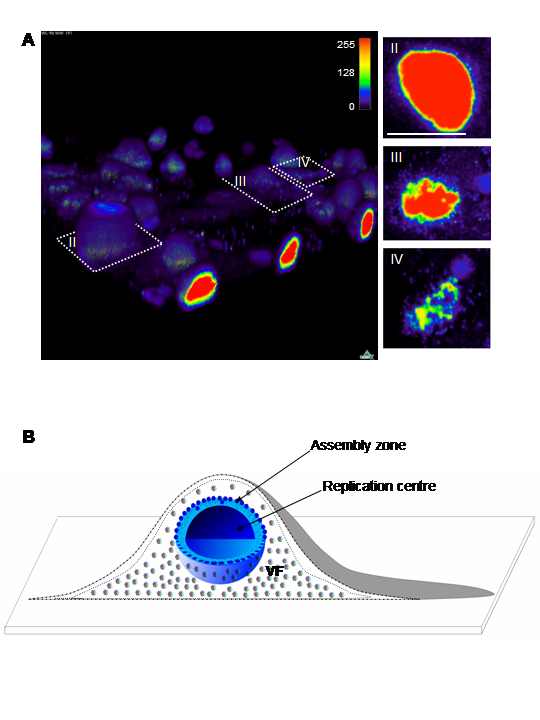

Supplement: Figure S2 — 3D reconstruction and model of Mimivirus factory. (A) Volumic reconstruction of Mimivirus factory. DAPI stained Mimivirus infected A. polyphaga were observed with a confocal microscope at 16 h p.i. Fluorescence intensity was represented by a rainbow logarithmic look up table. The respective 2D maximum intensity projections of the II, III and IV regions are shown in the lower part : II, mature stage of the growing VF; III, productive stage; IV, degenerative stage. Bar = 10 µm. (B) 3D model of Mimivirus factory. (0.31 MB TIF) [file pone.0000328.s004.tif]

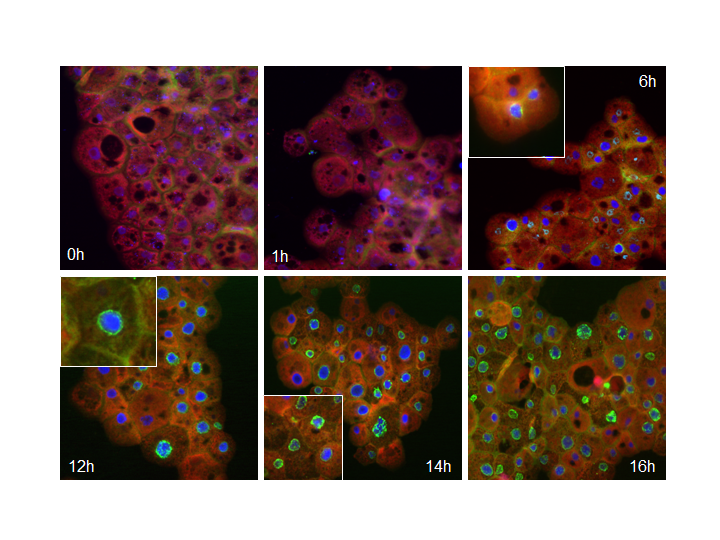

Supplement: Figure S3 — Molecular characterization of the Mimivirus factory. Combined labelling of Mimivirus AT-rich DNA with DAPI staining (direct fluorescence; blue) and Mimivirus R710 protein with a specific mAb by indirect immunofluorescence (green) was performed during the time course of A. polyphaga infection. The protein showed a punctuated staining pattern starting at 6 h post-infection around the DAPI-stained Mimivirus factory. Thereafter, the number and intensity of anti-R710 mAb-stained Mimivirus factory increased until the end of the viral cycle. (0.76 MB TIF) [file pone.0000328.s005.tif]
